# In Caring for Older People in Low- and Middle-Income Countries, Do Older Caregivers Have a High Level of Care Burden and Psychological Morbidity Compared to Younger Caregivers?

**DOI:** 10.3390/ijerph192416405

**Published:** 2022-12-07

**Authors:** Ruttana Phetsitong, Patama Vapattanawong, Rosie Mayston, Martin Prince, Kia-Chong Chua

**Affiliations:** 1 Faculty of Physical Therapy, Mahidol University, Nakhon Pathom 73170, Thailand; 2 Institute for Population and Social Research, Mahidol University, Nakhon Pathom 73170, Thailand; 3 Department of Global Health and Social Medicine, Social Science and Public Policy, King’s College London, London WC2R 2LS, UK; 4 King’s Global Health Institute, King’s College London, London WC2R 2LS, UK; 5 Institute of Psychiatry, Psychology and Neuroscience, King’s College London, London SE5 8AF, UK

**Keywords:** aging, caregiver, care burden, psychological morbidity, low- and middle-income countries

## Abstract

Caregivers have become older as longevity increases. Caregiving for older people can cause burdens and psychological morbidity, which are the chronic stresses perceived by informal caregivers. This study aimed to compare the levels of care burden and psychological morbidity between older and younger caregivers in low- and middle-income countries. A cross-sectional survey was conducted in Cuba, the Dominican Republic, Peru, Venezuela, Mexico, Puerto Rico, and China. Data were collected by the 10/66 Dementia Research Group. The Zarit Burden Inventory was used to measure the levels of burden on caregivers. Psychological morbidity was assessed through the Self-Reporting Questionnaire. Data from 1348 households in which informal caregivers provided home care for one older person were included in the analysis. Multivariable logistic regression was used to investigate the effects of caregiver age upon care burden and psychological morbidity. A fixed-effect meta-analysis model was used to obtain a pooled estimate of the overall odds ratios of each country. The unadjusted and the adjusted model for potential covariates revealed no significant difference in care burden and psychological morbidity between older caregivers and younger caregivers. The adjusted pooled estimates, however, indicated a lower psychological morbidity among older caregivers (OR = 0.61, 95% CI: 0.41–0.93, I^2^ = 0.0%). The demographic implications of caregiver age may suggest different policy responses across low- and middle-income countries.

## 1. Introduction

The aging population is growing in low- and middle-income countries (LMICs), along with an increase in comorbidities and caregiving needs [1,2]. Several demographic trends, such as low fertility, rising life expectancy, changing family structures, and marital patterns, have impacted the trend in caregiving (younger versus older caregivers). According to the demographic definition of age and concepts of development of life stage, a young caregiver is between the age of 15 years and before retirement. She/he is normally in middle age. It is a period in which many transitions take place. It is a time of schooling or parenthood. It may also be time to care for old parents; whereas, an older caregiver is usually of retirement age. At this age, they face more health issues and become aware of their mortality [3,4].

Care burden and psychological morbidity are the chronic stresses perceived by informal caregivers [5]. It is evident that half of caregivers experience burdens [6]. This is at the same level of prevalence of psychological distress [7,8]. A high level of caregiver burden and psychological morbidity results in sleep disturbances and depression, affecting quality of life [9]. Moreover, these conditions have been recognized as determinants of mortality for both the caregiver and the care receiver, and more so if the caregiver is older [10].

Psychological challenges have been reported by older caregivers regardless of their financial and economic stability [11,12]. In addition, older caregivers devote longer hours to caregiving than younger caregivers based on their perceived readiness and better preparedness [13,14]. Furthermore, the closer the relationship between the caregiver and the care receiver, the better the outcome, such as compromise, problem-solving skills, and positive adjustment [15]. Care burden and psychological morbidity are the results of a complex interaction between caregivers’ demographic backgrounds and the health conditions of the people for whom they are providing care [16,17]. Based largely on the theory of caregiving stress and the psychological wellbeing of caregivers [18,19], in addition to the caregiver’s age, sex is also a significant predictor of stress and burden. Female caregivers are more likely to experience social inequality because of their culturally-prescribed caring role. Therefore, female caregivers tend to report more burden than males [20]. Older people’s cognitive impairment has been repeatedly mentioned in the literature [21,22,23]. In addition to caregivers’ individual factors and older people’s health impairments, household characteristics have also been cited as confounding factors of caregiving stress [24].

The impact of caregiver age on their care burden and psychological morbidity might differ across cultures in LMICs, particularly Cuba, the Dominican Republic, Peru, Venezuela, Mexico, Puerto Rico, and China, due to social expectations and the lack of available long-term care by the state. In these countries, invisible burdens and mental problems are placed on informal caregivers. The caregivers often find caregiving quite challenging and feel conflicted about their responsibility [25]. Studies based on the 10/66 Dementia Research Group (DRG) survey of dementia and aging in LMICs have provided evidence that caring for older dependent people is associated with significant mental health problems in caregivers [26,27,28]. Previous studies have not focused on caregiver age and have not provided a multi-country analysis.

Therefore, this study aimed to compare the level of care burden and psychological morbidity between older and younger caregivers in Cuba, the Dominican Republic, Peru, Venezuela, Mexico, Puerto Rico, and China, in order to address the gap in the current literature on caregiving in the context of the 10/66 DRG surveys [29]. Our research question was as follows: Do older caregivers have a higher level of care burden and psychological morbidity compared to younger caregivers?

## 2. Materials and Methods

### 2.1. Study Design, Setting, and Sample

The study was a secondary analysis of data collected by the 10/66 DRG, with a population-based cohort design. The cohort study was carried out by means of a series of repeated cross-sectional surveys of older people living with dementia in low- and middle-income countries to better understand their health status [30]. Three waves of data covering Latin American countries (Cuba, the Dominican Republic, Peru, Argentina, Brazil, Venezuela, Mexico, and Puerto Rico), China, and India were collected. The first wave (prevalence phase) was aimed at measuring the prevalence of dementia and care arrangements, and evaluating the impact of the provision of care on caregivers in all the countries. The second wave (incidence phase) was a follow-up of the prevalence phase (India was excluded). The third wave used an extended version of the 10/66 DRG survey to ensure that the vital status of each participant was confirmed in Cuba, the Dominican Republic, Peru, Venezuela, Mexico, Puerto Rico, and China.

The survey was conducted with all residents ≥65 years of age and their caregivers, living in thirteen geographically defined rural and urban catchment area sites. Rural catchments were sparsely populated and featured lifestyles pertaining to traditional agriculture. Urban catchments were selected to include households with low socioeconomic status. Households with a predominantly middle-class or professional population with high incomes were excluded. Details of the methodology and protocol of the 10/66 DRG study have been described elsewhere [31]. A full assessment was performed of all eligible participants and included: socio-demographic background, health, and risk factors interview; a structured clinical mental state assessment; and a physical examination. The specific details of the data of each country have been published elsewhere [30].

To compare the findings of this study with longitudinal analyses of the effect of caregiver age on care burden and psychological morbidity in the future, this study used the first wave of data obtained from Cuba, the Dominican Republic, Peru, Venezuela, Mexico, Puerto Rico, and China. The survey was conducted between February 2003 and June 2009 (between 2003–2006 for Cuba; between 2003–2004 for the Dominican Republic; between 2005–2007 for Peru; between 2004–2006 for Venezuela; between 2005–2007 for Mexico; between 2003–2005 for China; and between 2006–2009 for Puerto Rico). The total sample of households with people ≥65 years of age from the seven countries was 11,717.

### 2.2. Selected Households, and Participants

Of the 11,717 households, 1560 were identified as having caregivers. Of these 1560 households, 1456 were considered to have a primary informal caregiver, defined as the person giving the most hands-on care or a family member acting as the main organizational care [32]. However, this study excluded households in which the primary caregiver provided care for more than one older person, regardless of whether the persons were diagnosed with or without dementia. Ultimately, the final data analysis included 1348 households. In order to test the research hypotheses, the selected households were classified into two groups: younger caregiver households (≤64 years of age) and older caregiver households (≥65 years of age).

### 2.3. Care Burden

The Zarit Burden Interview (ZBI), which is one of the most widely used and cited instruments, was used to measure caregiver burden. It has been translated and validated, thereby allowing for international comparisons [32,33,34]. It was used in the 10/66 pilot studies for the listed countries in this study; strong psychometric properties and cultural relevance were found [26]. The ZBI consists of 22 items that include health, psychological wellbeing, social life, finances, and the relationship between the caregiver and the older person. Each item was evaluated on a 5-point Likert scale (see Table S1). Scores from 41 to 88 indicate ‘a high burden’ of care (coded as 1), while scores below 41 signify ‘a low burden’ (coded as 0) [35,36].

### 2.4. Psychological Morbidity

This was assessed using the Self-Reporting Questionnaire (SRQ) which has been widely used in different populations [37]. The questionnaire includes 20 items, screening symptoms of depression, anxiety, and distress over the past two weeks (see Table S2). It yields a total score that ranges from 0 to 20. As recommended by the World Health Organization (WHO) [38], higher scores indicate higher psychological morbidity with a cut-off score of ≥8 were coded as 1, whereas scores of <8 were coded as 0 for non-psychological morbidity.

### 2.5. Covariates

Guided by the Pearlin Stress Process Model [18] and the Yates Stress Process Model [19], the study controlled for potential factors, including household characteristics, health status, the caregiver’s socio-demographic status, and hours dedicated to caregiving. These were entered into the model analyses as potential confounding factors, since these variables might affect the caregiver’s care burden and psychological morbidity.

Considering household characteristics, the size of a household is often associated with considerable mental health issues due to long-term care for older people in the household [24]. In this study, household size referred to the number of household members in the older person’s household. In addition, household assets were a proxy for the socio-economic status of the household, since this represents a more permanent status than either income or consumption [39]. The number of assets in a household was measured based on seven possible total lists (television, fridge/freezer, water mains, electricity, telephone, plumbed toilet, and plumbed bathroom).

Dementia/cognitive impairment, behavioral challenges, and functional dependency have been frequently reported in the literature as components of caregiver stress. In our study, cognitive impairment was measured either by the three validated 10/66 dementia diagnostic assessments or the dementia criterion of the Diagnostic and Statistical Manual of Mental Disorders (DSM-IV). The three 10/66 assessments included the Consortium to Establish a Registry for Alzheimer’s Disease (CERAD) 10-word list recall task, the Community Screening Instrument for Dementia (CSI-D), and the Geriatric Mental State examination (GMS) [31,40].

Functional dependency was defined as difficulty in performing basic activities of daily living (ADLs), such as eating, dressing, toileting, bathing, and walking, as assessed by the disability assessment schedule (WHO-DAS) developed by the World Health Organization [41]. This measure has shown promise in predicting the disability level of older people, which then affects care burden [42]. Severe difficulty with ≥3 basic ADLs was coded as 1, and severe difficulty with <3 ADLs was coded as 0.

Twelve behavioral characteristics were identified based on their severity and included delusions, hallucinations, agitation/aggression, depression, anxiety, irrational euphoria, apathy, disinhibition, irritability, motor disturbance, disruptive nighttime behavior, and adverse appetite/eating changes. Measurement was based on the Neuropsychiatric Inventory-Questionnaire (12NPI-Q) score, which was administered to the primary caregiver [43].

Additional variables that have been frequently reported regarding the caregiver’s background include sex, marital status, and education level [20,44,45,46]. In this study, marital status was coded as a partnership (currently married or cohabiting) or as a non-partnership (single/widowed/divorced/separated). As for education, caregivers with lower education reported a higher burden in terms of caregiving [46]. In this study, education was categorized into primary or lower, and higher than primary.

Caregiving mental health may be influenced not only by the health status of the person being cared for, but also by the amount of time (in hours) dictated for caregiving. This is usually reported as the hours spent a day assisting with ADLs (eating, dressing, toileting, bathing, communicating, using transportation, looking after one’s appearance, and supervising). Therefore, longer hours may lead to greater health challenges [47,48].

### 2.6. Statistical Methods

Descriptive analysis was employed to describe the sample, analyze the distribution of the background variables, and explore the quantitative level of the variables. The Kolmogorov–Smirnov normality test was used to assess the normality of the distributions. The independent sample t-test was used to compare the mean differences between the two study groups if the data showed normal distribution. However, if the data were not normally distributed, a Mann–Whitney U test was applied to compare median differences of covariate variables (household sizes, the number of household assets, the behavioral problem scores, and caregiving hours) between two caregiver age groups. The chi-squared test was used to compare the proportion of covariate variables in each caregiver age group: dementia, functional dependency, caregiver sex, caregiver marital status, and caregiver educational levels [49].

There were two stages of analysis used to investigate the effects of the independent variable (caregiver age group) upon the dichotomous outcome variables (care burden and psychological morbidity). In the first stage, binary logistic regression models were applied for each country. The model analyses were performed for unadjusted and adjusted models. All covariate variables (household size, number of household assets, caregiver sex, caregiver marital status, caregiver education, cognitive impairment of older person, functional dependency of older person, behavioral problems of older person, and caregiving hours) were controlled in the adjusted model. The second stage in this study collected an odds ratio (OR) and 95% confidence interval (CI) for each country, and looked for the overall average magnitude of the OR and 95% CI of caregiver age on care burden and psychological morbidity using an inverse-variance weighted (fixed-effect) meta-analysis. All the statistical analyses were performed using STATA/SE 14.0 [50].

## 3. Results

### 3.1. Sample Characteristics

A total of 1348 households were included in this analysis. Across the seven countries, the percentage of households with older caregivers was lower compared to those with younger caregivers. Puerto Rico had the highest percentage of households with older caregivers (45.2%). The lowest percentage (12.2%) was found in Venezuela. The percentage of older caregivers in China (40.2%) was higher than that of Cuba (33.3%), Peru (24.8%), the Dominica Republic (23.1%), and Mexico (19.7%). Table 1 and Table 2 summarize the characteristics and the background variables.

Our findings revealed that household background and the health status of older people were similar among younger and older caregivers in each country. As compared across countries, the average household size of Puerto Rico was the smallest (median = 2, 25th percentile = 2, and 75th percentile = 3). There were fewer behavioral problems among older people in China (Table 1).

Most caregivers were female; whereas, a statistical difference was noticed in caregiver gender and age in Peru, Mexico, and Puerto Rico. Across all countries, young and older caregiver households had a higher prevalence of partnership caregivers, except in Puerto Rico (88.2% of older caregivers and 73.8% of younger caregivers were single/widowed/divorced/separated). In terms of caregiver education, the chi-square test indicated that there were significant differences between younger and older caregivers across the seven countries. It is not surprising that younger caregivers have a higher education level than older caregivers. However, the majority of older caregivers in Cuba, Peru, and Puerto Rico had an educational level higher than the primary level (Table 2).

Notably, the median number of hours delivering care was different across countries; in Venezuela, only one hour a day was dedicated. However, there was no significant difference in time between the older and younger caregivers (Table 2).

### 3.2. The Effects of Caregiver Age upon Care Burden and Psychological Morbidity

A multivariable model based on binary logistic regression analysis was used to investigate the effect of caregiver age group (older caregivers vs. younger caregivers) upon their care burden and psychological morbidity, along with other potential confounders, as shown in Table 3.

In model I, the unadjusted analysis showed no significant differences in care burden and psychological morbidity between older and younger caregivers across the seven countries. The pooled estimates OR and 95% CI remained unaffected (OR = 0.84, 95% CI 0.58–1.24, I^2^ = 0.0% for care burden, *p*-value = 0.39; OR = 0.77, 95% CI 0.56–1.06, I^2^ = 3.7%, *p*-value = 0.11 for psychological morbidity).

Likewise, the adjusted analysis in model II showed no statistically significant differences in caregiver burden and psychological morbidity between younger and older caregivers in all seven countries. However, the pooled estimates showed that psychological morbidity was significantly associated with caregiver age (OR = 0.61, 95% CI 0.41–0.93, I^2^ = 0.0%), with older caregivers at 39% less risk of psychological morbidity than younger caregivers. It is worth noting that the odds ratio of psychological morbidity (the unadjusted model, OR = 6.21; the adjusted model, OR = 5.22) in China was surprisingly high, although this was not significant.

## 4. Discussion

This study investigated the association between caregiver age and their care burden and psychological morbidity. Overall, the findings of the multivariable analysis indicated that, across all countries, older caregivers did not report a higher burden and psychological morbidity. The findings for Cuba and the Dominican Republic are in accordance with previous studies on the burden and psychological distress of caregiving [51,52]. Familism is a fundamental value of Latino cultures; it is cited as a motivating factor for providing care for members of the same family. Both younger and older caregivers in Cuba and the Dominican Republic had strong family stability, obligations, and openness about who should provide care for family members [51,52]. Similarly, Puerto Rican and Mexican caregivers perceived a greater sense of duty toward older members in terms of their care at any age [53,54]. However, a previous study in Peru found that caregivers aged ≥65 years perceived less care burden than those <65 [55]. This may be because a second caregiver was available for the older caregiver in the previous study. In the case of Asian cultures, they have a strong sense of responsibility toward older family members. The results of the analysis in China are consistent with a prior study in Thailand, with no association being found between the caregiver age group and psychological burden in older people’s households [56]. It may be related to principles of filial piety, which are important to older individuals and regarded as a necessary component of fulfillment in older age.

It should be noted that the odds ratio for psychological morbidity in China was surprisingly high. A large odds ratio with wide confidence intervals usually occurs when the prevalence of an event of interest is very low in a sample [57]. The sample of Chinese caregivers with psychological morbidity was extremely small in this study (see Table S3). As a result, the statistical power to estimate with precision is undermined, leading to overestimates of the odds ratio and very wide confidence intervals. This may also have affected the Mexican care burden data (see Table S3).

Although age did not have a significant effect on care burden and psychological morbidity, the pooled estimates of the multivariate analysis for all countries revealed that the risk of psychological morbidity was lower in older caregivers than in younger caregivers. The possible explanation for this is that caregivers may have different issues depending on their age. The availability and preparedness of older age might be related to more caregiving effectiveness, and less caregiving stress and burden [5]. Multiple daily stressors lead to overwhelming stress and psychological morbidity in caregivers with multiple responsibilities [8].

In addition to caregiving burdens and psychological morbidity, younger caregivers are often in the midst of transitions into careers and/or parenthood. Becoming a caregiver for their older members can impact a young adult’s ability to form intimate relationships, engage in family life, or obtain career goals [58]. Maintaining a career while providing care has been associated with strain, and young adult caregivers have reported difficulties in managing both responsibilities [58,59]. Our analysis showed that younger caregivers had more children (≤16 years of age) compared to older ones (see Table S4). Therefore, amplified psychological stress may stem from a lack of personal resources (time and energy) and career disruption [60,61].

Based on the caregiving over life course theory, old age is a time in which many roles are lost owing to widowhood, retirement, the death of friends, and the limitations imposed by illnesses. Therefore, the caregiving role is a normative or, at least, expected feature of this stage of life as opposed to midlife [62,63]. Apart from fewer multiple-role demands and fewer time constraints, older caregivers have a lower psychological morbidity than younger caregivers due to more personal satisfaction [14]. The developmental stage of older age usually involves a transition to retirement, and the goals of work performance and achievement typically shift to finding goals that afford one a sense of inner fulfillment and that maintain valued relationships [61]. A large proportion of older caregivers in this study were providing care to their spouses. In these relationships, the care is often mutual, with a couple working as a team to care for each other.

A major strength of our study was the multi-country analysis that examined the effect of caregiver age on care burden and psychological morbidity across many cultures. However, we detected several limitations. First, the results from a cross-sectional survey were unable to evaluate causality. The prior mental health status of the caregivers or how this may change over time cannot be determined. Second, we only included households where care was provided to only one older person. Thus, a small number of households in which caregivers care for more than one older person were not included. Third, there were no available data on physical health status and social support for caregivers that should be considered mediators for adjustment of the care burden and psychological morbidity. Fourth, this study focused on data from the first wave of the 10/66 study, which may be out of date. However, this might provide baseline information for further research. Lastly, the study used a quantitative approach to enable a comparison of findings across countries. In order to have a more complete analysis, future studies should consider a qualitative approach. In-depth interviews with caregivers and care receivers may help uncover the foundation of the burden and psychological morbidity. Further work is required to explore the psychological morbidity among younger caregivers who provide care to older people and to identify factors that affect psychological morbidity in order to obtain suggestions for improvement of the mental health of caregivers of older people in LMICs.

## 5. Conclusions

The distribution of households with older caregivers was inconsistent across the seven LMICs. However, the percentage of households with older caregivers was almost equal to that of households with younger caregivers in Puerto Rico and China. The extent of care burden between older and younger caregivers was not different. However, older caregivers were at a lower risk of psychological morbidity than younger caregivers. In different countries, the rate at which the population is aging substantially varies. Considering the demographic implications of caregiver age may suggest different policy responses across LMICs. Since many of the caregivers are from the younger population with multiple roles, they would most likely need additional assistance with caregiving. It is possible that governments will find some implications from this study, including providing credit or a caregiver benefit for younger caregivers in the social security system. The findings of this study may also shift research prioritization to younger caregivers in Latin America and China.

## Figures and Tables

**Table 1:** Percentage of caregiver’s age group and the background characteristics of households and older people in seven countries (*n* = 1348 households).

			Household Characteristics		Older People’s Health Status				
			Size	Number of Assets	Dementia		Functional Dependency		Behavioral Problem
		(%)	Median (p25, p75)		No (%)	Yes (%)	No (%)	Yes (%)	Median (p25, p75)
Cuba (*n* = 222)	O	33.3	3 (2, 4)	6 (5, 6)	29.7	70.3	39.2	60.8	4 (2, 8)
	Y	66.7	3 (2, 5)	6 (5, 6)	27.7	72.3	39.2	60.8	5 (2, 10)
	*p*-value		ns	ns	ns		ns		ns
Dominican Republic (*n* = 208)	O	23.1	3 (2, 5)	5 (4, 6)	52.1	47.9	54.2	45.8	6 (3, 10)
	Y	76.9	4 (3, 6)	5 (4, 6)	54.4	45.6	60.6	39.4	8 (3, 15)
	*p*-value		ns	ns	ns		ns		ns
Peru (*n* = 137)	O	24.8	5 (3, 7)	6 (6, 6)	52.9	47.1	41.2	58.8	5 (2, 10)
	Y	75.2	4 (3, 6)	6 (6, 6)	47.6	52.4	55.3	44.7	5 (2, 9)
	*p*-value		ns	ns	ns		ns		ns
Venezuela (*n* = 181)	O	12.2	4 (2, 5)	6 (6, 6)	45.5	54.5	72.7	27.3	7 (4, 13)
	Y	87.8	4 (3, 6)	6 (6, 7)	51.6	48.4	78.6	21.4	4 (1, 8)
	*p*-value		ns	ns	ns		ns		ns
Mexico (*n* = 157)	O	19.7	2 (1, 4)	5 (4, 6)	71.0	29.0	58.1	41.9	4 (1, 6)
	Y	80.3	3 (1, 5)	6 (5, 6)	58.7	41.3	71.4	28.6	5 (2, 9)
	*p*-value		ns	ns	ns		ns		ns
Puerto Rico (*n* = 239)	O	45.2	2 (2, 3)	7 (6, 7)	42.1	57.9	47.2	52.8	3 (0, 6)
	Y	54.8	2 (2, 3)	7 (6, 7)	44.6	55.4	44.3	55.7	3 (0, 8)
	*p*-value		ns	ns	ns		ns		ns
China (*n* = 204)	O	40.2	3 (2, 5)	5 (5, 6)	57.3	42.7	48.8	51.2	0 (0, 1)
	Y	59.8	3 (2, 4)	6 (5, 7)	55.7	44.3	54.1	45.9	0 (0, 2)
	*p*-value		ns	ns	ns		ns		ns

Note: O = older caregiver; Y = younger caregiver; p25 = the 25th percentile; p75 = the 75th percentile; ns = not significant.

**Table 2:** Socio-demographic backgrounds and caregiving hours among caregivers in seven countries (*n* = 1348 households).

		Caregiver Characteristics						Caregiving Hours
		Sex		Marital Status		Education		
		Male (%)	Female (%)	P (%)	NP (%)	P (%)	S (%)	Median (p25, p75)
Cuba (*n* = 222)	O	23.0	77.0	69.0	31.0	47.3	52.7	5 (2, 9)
	Y	18.9	81.1	62.6	37.4	7.4	92.6	5 (2, 7)
	*p*-value	ns		ns		***		ns
Dominican Republic (*n* = 208)	O	20.8	79.2	71.1	28.9	87.5	12.5	3 (1, 5)
	Y	18.8	81.2	50.8	49.2	44.0	56.0	4 (1, 6)
	*p*-value	ns		*		***		ns
Peru (*n* = 137)	O	28.2	61.8	76.5	23.5	44.1	55.9	6 (4, 6)
	Y	8.7	91.3	42.7	57.3	20.4	79.6	6 (2, 7)
	*p*-value	***		***		**		ns
Venezuela (*n* = 181)	O	27.3	72.7	50.0	50.0	59.1	40.9	1 (0, 3)
	Y	17.8	82.2	46.8	53.2	27.7	72.3	1 (0, 3)
	*p*-value	ns		ns		***		ns
Mexico (*n* = 157)	O	41.9	58.1	71.0	29.0	87.1	12.9	3 (1, 5)
	Y	14.3	85.7	68.3	31.8	38.9	61.1	3 (1, 5)
	*p*-value	***		ns		***		ns
Puerto Rico (*n* = 239)	O	35.2	64.8	11.8	88.2	39.8	60.2	4 (1, 6)
	Y	16.2	83.2	26.2	73.8	17.6	82.4	3 (1, 5)
	*p*-value	***		**		***		ns
China (*n* = 204)	O	42.7	57.3	97.6	2.4	61.0	39.0	5 (2, 8)
	Y	34.4	65.6	91.0	9.0	27.9	72.1	4 (1, 8)
	*p*-value	ns		ns		***		ns

Note: O = older caregiver; Y = younger caregiver; p25 = the 25th percentile; p75 = the 75th percentile; Marital status (P = partnership, NP = non-partnership); Education (P = primary or less, S = higher than primary); *** *p* < 0.001; ** *p* < 0.01; and * *p* < 0.05; ns = not significant.

**Table 3:** Unadjusted and adjusted association between older caregiver and caregiver care burden and psychological morbidity.

Country	Care Burden					Psychological Morbidity				
	*n*	OR	95% CI		*p*-Value	*n*	OR	95% CI		*p*-Value
			Lower	Upper				Lower	Upper	
**Model I** (unadjusted model)										
Cuba	220	1.12	0.57	2.23	0.74	222	0.60	0.30	1.20	0.15
Dominican Republic	206	0.80	0.28	2.27	0.68	208	0.85	0.41	1.78	0.67
Peru	137	0.94	0.32	2.78	0.91	137	0.78	0.36	1.69	0.52
Venezuela	178	1.43	0.44	4.63	0.55	181	1.50	0.51	4.44	0.46
Mexico	156	0.43	0.05	3.52	0.43	157	0.52	0.18	1.46	0.22
Puerto Rico	239	0.38	0.12	1.22	0.10	239	0.65	0.33	1.30	0.23
China	204	0.67	0.27	1.63	0.38	204	6.21	0.68	56.55	0.11
Pooled estimate		0.84	0.58	1.24	0.39		0.77	0.56	1.06	0.11
Heterogeneity I^2^		0.0%					3.7%			
**Model II** (adjusted model)										
Cuba	206	1.17	0.50	2.76	0.72	208	0.48	0.20	1.14	0.09
Dominican Republic	173	1.30	0.34	4.91	0.70	175	0.82	0.33	2.05	0.67
Peru	134	0.23	0.03	1.41	0.11	134	0.45	0.14	1.41	0.17
Venezuela	159	0.72	0.09	5.49	0.75	162	1.24	0.29	5.24	0.77
Mexico	122	2.59	0.18	36.95	0.48	152	0.35	0.09	1.28	0.11
Puerto Rico	202	0.29	0.07	1.18	0.08	202	0.53	0.22	1.24	0.14
China	202	0.41	0.12	1.34	0.14	189	5.22	0.51	53.94	0.17
Pooled estimate		0.73	0.43	1.21	0.22		0.61 *	0.41	0.93	0.02
Heterogeneity I^2^		9.9%					0.0%			

Note: Model II adjusted for sex, marital status, education of caregivers, cognitive impairment, functional dependency, behavioral problem of the older person, time spent helping with ADLs, household size, and number of household assets; OR = odds ratio; 95CI = 95% confidence interval; * *p* < 0.05.

## Data Availability

Data are available from the 10/66 Dementia Research Group (https://1066.alzint.org/ (accessed on 29 October 2022)) upon reasonable request.

## Supplementary Materials

The following supporting information can be downloaded at: https://www.mdpi.com/article/10.3390/ijerph192416405/s1, Table S1: The Zarit Burden Interview (ZBI); Table S2: Self-reporting questionnaire (SRQ); Table S3: Number of caregivers according to care burden and psychological morbidity by country; Table S4: Cross-tabulation between the number of children (aged under 16 years) in a household and caregiver age.

## References

[1] Abegunde D.O., Mathers C.D., Adam T., Ortegon M., Strong K. (2007). The burden and costs of chronic diseases in low-income and middle-income countries. Lancet.

[2] World Health Organization (2005). Facing the Facts: Chronic Diseases in Low and Middle Income Countries.

[3] King E.M., Randolph H.L., Floro M.S., Suh J. (2021). Demographic, health, and economic transitions and the future care burden. World Dev..

[4] Hareven T.K. (2018). Aging and Generational Relations: A Historical and Life-Course Perspective. Families, History, and Social Change.

[5] Schulz R., Sherwood P.R. (2008). Physical and mental health effects of family caregiving. J. Soc. Work Educ..

[6] Faronbi J.O., Olaogun A.A. (2017). The influence of caregivers’ burden on the quality of life for caregivers of older adults with chronic illness in Nigeria. Int. Psychogeriatr..

[7] Sambasivam R., Liu J., Vaingankar J.A., Ong H.L., Tan M.E., Fauziana R., Picco L., Chong S.A., Subramaniam M. (2019). The hidden patient: Chronic physical morbidity, psychological distress, and quality of life in caregivers of older adults. Psychogeriatrics.

[8] Hudson P., Trauer T., Kelly B., O’Connor M., Thomas K., Summers M., Zordan R., White V. (2013). Reducing the psychological distress of family caregivers of home-based palliative care patients: Short-term effects from a randomised controlled trial. Psychooncology.

[9] Liu S., Li C., Shi Z., Wang X., Zhou Y., Liu S., Liu J., Yu T., Ji Y. (2017). Caregiver burden and prevalence of depression, anxiety and sleep disturbances in Alzheimer’s disease caregivers in China. J. Clin. Nurs..

[10] Garlo K., O’Leary J.R., Van Ness P.H., Fried T.R. (2010). Burden in caregivers of older adults with advanced illness. J. Am. Geriatr. Soc..

[11] Schulz R., O’Brien A.T., Bookwala J., Fleissner K. (1995). Psychiatric and physical morbidity effects of dementia caregiving: Prevalence, correlates, and causes. Gerontologist.

[12] Donaldson C., Tarrier N., Burns A. (1997). The impact of the symptoms of dementia on caregivers. Br. J. Psychiatry.

[13] King McLaughlin J., Greenfield J.C., Hasche L., De Fries C. (2019). Young adult caregiver strain and benefits. Soc. Work Res..

[14] Archbold P.G., Stewart B.J., Greenlick M.R., Harvath T. (1990). Mutuality and preparedness as predictors of caregiver role strain. Res. Nurs. Health.

[15] Fauth E., Hess K., Piercy K., Norton M., Corcoran C., Rabins P., Lyketsos C., Tschanz J. (2012). Caregivers’ relationship closeness with the person with dementia predicts both positive and negative outcomes for caregivers’ physical health and psychological well-being. Aging Ment. Health.

[16] Areia N.P., Fonseca G., Major S., Relvas A.P. (2019). Psychological morbidity in family caregivers of people living with terminal cancer: Prevalence and predictors. Palliat. Support. Care.

[17] Zarit S.H., Reever K.E., Bach-Peterson J. (1980). Relatives of the impaired elderly: Correlates of feelings of burden. Gerontologist.

[18] Pearlin L.I., Mullan J.T., Semple S.J., Skaff M.M. (1990). Caregiving and the stress process: An overview of concepts and their measures. Gerontologist.

[19] Yates M.E., Tennstedt S., Chang B.-H. (1999). Contributors to and mediators of psychological well-being for informal caregivers. J. Gerontol. B Psychol. Sci. Soc. Sci..

[20] Etters L., Goodall D., Harrison B.E. (2008). Caregiver burden among dementia patient caregivers: A review of the literature. J. Am. Acad. Nurse Pract..

[21] Domingues N.S., Verreault P., Hudon C. (2018). Reducing burden for caregivers of older adults with mild cognitive impairment: A systematic review. Am. J. Alzheimers Dis. Other Demen..

[22] Garand L., Amanda Dew M., Eazor L.R., DeKosky S.T., Reynolds III C.F. (2005). Caregiving burden and psychiatric morbidity in spouses of persons with mild cognitive impairment. Int. J. Geriatr. Psychiatry.

[23] González-Salvador M.T., Arango C., Lyketsos C.G., Barba A.C. (1999). The stress and psychological morbidity of the Alzheimer patient caregiver. Int. J. Geriatr. Psychiatry.

[24] Mayston R., Guerra M., Huang Y., Sosa A.L., Uwakwe R., Acosta I., Ezeah P., Gallardo S., de Oca V.M., Wang H. (2014). Exploring the economic and social effects of care dependence in later life: Protocol for the 10/66 research group INDEP study. Springerplus.

[25] Mayston R., Lloyd-Sherlock P., Gallardo S., Wang H., Huang Y., Montes de Oca V., Ezeah P., Guerra M., Sosa A.L., Liu Z. (2017). A journey without maps—Understanding the costs of caring for dependent older people in Nigeria, China, Mexico and Peru. PLoS ONE.

[26] Prince M. (2004). Care arrangements for people with dementia in developing countries. Int. J. Geriatr. Psychiatry.

[27] Brinda E.M., Rajkumar A.P., Enemark U., Attermann J., Jacob K. (2014). Cost and burden of informal caregiving of dependent older people in a rural Indian community. BMC Health Serv. Res..

[28] Prince M., Brodaty H., Uwakwe R., Acosta D., Ferri C.P., Guerra M., Huang Y., Jacob K., Llibre Rodriguez J.J., Salas A. (2012). Strain and its correlates among carers of people with dementia in low-income and middle-income countries. A 10/66 Dementia Research Group population-based survey. Int. J. Geriatr. Psychiatry.

[29] Prina A.M., Mayston R., Wu Y.-T., Prince M. (2019). A review of the 10/66 dementia research group. Soc. Psychiatry Psychiatr. Epidemiol..

[30] Prina A.M., Acosta D., Acosta I., Guerra M., Huang Y., Jotheeswaran A., Jimenez-Velazquez I.Z., Liu Z., Llibre Rodriguez J.J., Salas A. (2017). Cohort profile: The 10/66 study. Int. J. Epidemiol..

[31] Prince M., Ferri C.P., Acosta D., Albanese E., Arizaga R., Dewey M., Gavrilova S.I., Guerra M., Huang Y., Jacob K. (2007). The protocols for the 10/66 dementia research group population-based research programme. BMC Public Health.

[32] Van Durme T., Macq J., Jeanmart C., Gobert M. (2012). Tools for measuring the impact of informal caregiving of the elderly: A literature review. Int. J. Nurs. Stud..

[33] Chou K.-R., Chu H., Tseng C.-L., Lu R.-B. (2003). The measurement of caregiver burden. J. Med. Sci..

[34] Whalen K.J., Buchholz S.W. (2009). The reliability, validity and feasibility of tools used to screen for caregiver burden: A systematic review. JBI Evid. Synth..

[35] Zarit S., Orr N.K., Zarit J.M. (1985). The Hidden Victims of Alzheimer’s Disease: Families under Stress.

[36] Hébert R., Bravo G., Préville M. (2000). Reliability, validity and reference values of the Zarit Burden Interview for assessing informal caregivers of community-dwelling older persons with dementia. Can. J. Aging/La Revue Canadienne du Vieillissement.

[37] Mari J.D.J., Williams P. (1985). A comparison of the validity of two psychiatric screening questionnaires (GHQ-12 and SRQ-20) in Brazil, using Relative Operating Characteristic (ROC) analysis. Psychol. Med..

[38] Beusenberg M., Orley J.H., World Health Organization (1994). A User’s Guide to the Self-Reporting Questionnaire (SRQ).

[39] Filmer D., Pritchett L.H. (2001). Estimating wealth effects without expenditure data—Or tears: An application to educational enrollments in states of India. Demography.

[40] Stewart R., Guerchet M., Prince M. (2016). Development of a brief assessment and algorithm for ascertaining dementia in low-income and middle-income countries: The 10/66 short dementia diagnostic schedule. BMJ Open.

[41] World Health Organization (1988). WHO Psychiatric Disability Assessment Schedule (WHO/DAS: With a Guide to Its Use.

[42] Clark D.O., Stump T.E., Tu W., Miller D.K. (2015). Improving the validity of activity of daily living dependency risk assessment. J. Appl. Gerontol..

[43] Kaufer D.I., Cummings J.L., Ketchel P., Smith V., MacMillan A., Shelley T., Lopez O.L., DeKosky S.T. (2000). Validation of the NPI-Q, a brief clinical form of the Neuropsychiatric Inventory. J. Neuropsychiatry Clin. Neurosci..

[44] Carlson D.L. (2012). Deviations from desired age at marriage: Mental health differences across marital status. J. Marriage Fam..

[45] Horn E.E., Xu Y., Beam C.R., Turkheimer E., Emery R.E. (2013). Accounting for the physical and mental health benefits of entry into marriage: A genetically informed study of selection and causation. J. Fam. Psychol..

[46] Montgomery R.J., Gonyea J.G., Hooyman N.R. (1985). Caregiving and the experience of subjective and objective burden. Fam Relat..

[47] Conlin M., Caranasos G., Davidson R. (1992). Reduction of caregiver stress by respite care: A pilot study. South. Med. J..

[48] McFall S., Miller B.H. (1992). Caregiver burden and nursing home admission of frail elderly persons. J. Gerontol..

[49] Du Prel J.-B., Röhrig B., Hommel G., Blettner M. (2010). Choosing statistical tests: Part 12 of a series on evaluation of scientific publications. Dtsch Arztebl Int..

[50] StataCorp LP (2015). StataCorp Stata Statistical Software: Release 14.

[51] Friedemann M.-L., Buckwalter K.C., Newman F.L., Mauro A.C. (2013). Patterns of caregiving of Cuban, other Hispanic, Caribbean black, and white elders in South Florida. J. Cross Cult. Gerontol..

[52] Medrano M., Rosario R.L., Payano A.N., Capellán N.R. (2014). Burden, anxiety and depression in caregivers of Alzheimer patients in the Dominican Republic. Dement. Neuropsychol..

[53] Ramos B.M. (2004). Culture, ethnicity, and caregiver stress among Puerto Ricans. J. Appl. Gerontol..

[54] Clark M., Huttlinger K. (1998). Elder care among Mexican American families. Clin. Nurs. Res..

[55] Custodio N., Lira D., Herrera-Perez E., Prado L.N.d., Parodi J., Guevara-Silva E., Castro-Suarez S., Mar M., Montesinos R., Cortijo P. (2014). Informal caregiver burden in middle-income countries Results from Memory Centers in Lima-Peru. Dement. Neuropsychol..

[56] Phetsitong R., Vapattanawong P., Sunpuwan M., Völker M. (2019). State of household need for caregivers and determinants of psychological burden among caregivers of older people in Thailand: An analysis from national surveys on older persons. PLoS ONE.

[57] Nemes S., Jonasson J.M., Genell A., Steineck G. (2009). Bias in odds ratios by logistic regression modelling and sample size. BMC Med. Res. Methodol..

[58] Dellmann-Jenkins M., Brittain L. (2003). Young adults’ attitudes toward filial responsibility and actual assistance to elderly family members. J. Appl. Gerontol..

[59] Dellmann-Jenkins M., Blankemeyer M., Pinkard O. (2001). Incorporating the elder caregiving role into the developmental tasks of young adulthood. Int. J. Aging Hum. Dev..

[60] Zarit S.H., Zarit J.M. (2015). Family caregiving. Psychology and Geriatrics.

[61] Harden J. (2005). Developmental life stage and couples’ experiences with prostate cancer: A review of the literature. Cancer Nurs..

[62] Pavalko E.K. (2011). Caregiving and the life course: Connecting the personal and the public. Handbook of Sociology of Aging.

[63] Moen P., Wethington E., Willis S.L., Reid J.D. (1999). Midlife development in a life course context. Life in the Middle.

