# Supplementary material for: In Caring for Older People in Low- and Middle-Income Countries, Do Older Caregivers Have a High Level of Care Burden and Psychological Morbidity Compared to Younger Caregivers?
Source: Int J Environ Res Public Health. 2022 Dec 7;19(24):16405. doi: 10.3390/ijerph192416405 (PMC9778435; doi:10.3390/ijerph192416405)
Supplement: Supplementary file 1 [file ijerph-19-16405-s001.zip › ijerph-2030817-supplementary.pdf]

Supplementary Table

Table S1. The Zarit Burden Interview (ZBI). Instructions: The following questions reflect how people sometimes feel when taking care of another person. After each question, indicate how often you feel that way, 0 = never, 1 = rarely, 2 = sometimes, 3 = quite frequently, or 4 = nearly always. There are no right or wrong answers.

**Question**	**Score**				
1. Do you feel that your relative asks for more help than he/she needs?	0	1	2	3	4
2. Do you feel that because of the time you spend with your relative that you do not have enough time for yourself?	0	1	2	3	4
3. Do you feel stressed between caring for your relative and trying to meet other responsibilities for your family or work?	0	1	2	3	4
4. Do you feel embarrassed over your relative's behaviour?	0	1	2	3	4
5. Do you feel angry when you are around your relative?	0	1	2	3	4
6. Do you feel that your relative currently affects your relationship with other family members or friends in a negative way?	0	1	2	3	4
7. Are you afraid what the future holds for your relative?	0	1	2	3	4
8. Do you feel your relative is dependent upon you?	0	1	2	3	4

9. Do you feel strained when you are around your relative?	0	1	2	3	4
10. Do you feel your health has suffered because of your involvement with your relative?	0	1	2	3	4
11. Do you feel that you do not have as much privacy as you would like, because of your relative?	0	1	2	3	4
12. Do you feel that your social life has suffered because you are caring for your relative?	0	1	2	3	4
13. Do you feel uncomfortable about having friends over, because of your relative?	0	1	2	3	4
14. Do you feel that your relative seems to expect you to take care of her/him, as if you were the only one she/he could depend on?	0	1	2	3	4
15. Do you feel that you do not have enough money to care for your relative, in addition to the rest of your expenses?	0	1	2	3	4
16. Do you feel that you will be unable to take care of your relative much longer?	0	1	2	3	4
17. Do you feel you have lost control of your life since your relative's illness?	0	1	2	3	4

18. Do you wish you could just leave the care of your relative to someone else?	0	1	2	3	4
19. Do you feel uncertain about what to do about your relative	0	1	2	3	4
20. Do you feel you should be doing more for your relative?	0	1	2	3	4
21. Do you feel you could do a better job in caring for your relative?	0	1	2	3	4
22. Overall, how burdened do you feel in caring for your relative?	0	1	2	3	4

Table S2. Self-reporting questionnaire (SRQ). Instructions: The following questions are related to certain pains and problems, that may have bothered you in the last 30 days. If you think the question applies to you and you had to describe the problem in the last 30 days, answer YES. On the other hand, if the question does not apply to you and you did not have the problem in the last 30 days, answer NO.

**Question**	**Response**	
1. Do you often have headaches?	No (0)	Yes (1)
2. Is your appetite poor?	No (0)	Yes (1)
3. Do you sleep badly?	No (0)	Yes (1)
4. Are you easily frightened?	No (0)	Yes (1)
5. Do your hands shake?	No (0)	Yes (1)
6. Do you feel nervous, tense or worried?	No (0)	Yes (1)

7. Is your digestion poor?	No (0)	Yes (1)
8. Do you have trouble thinking clearly?	No (0)	Yes (1)
9. Do you feel unhappy?	No (0)	Yes (1)
10. Do you cry more than usual?	No (0)	Yes (1)
11. Do you find it difficult to enjoy your daily activities?	No (0)	Yes (1)
12. Do you find it difficult to make decisions?	No (0)	Yes (1)
13. Is your daily work suffering?	No (0)	Yes (1)
14. Are you unable to play a useful part in life?	No (0)	Yes (1)
15. Have you lost interest in things?	No (0)	Yes (1)
16. Do you feel that you are a worthless person?	No (0)	Yes (1)
17. Has the thought of ending your life been in your mind?	No (0)	Yes (1)
18. Do you feel tired all the time?	No (0)	Yes (1)
19. Do you have uncomfortable feelings in your stomach	No (0)	Yes (1)
20. Are you easily tired?	No (0)	Yes (1)

Table S3. Number of caregivers according to care burden and psychological morbidity by country

	**Care burden**			**Psychological morbidity**		
	**n**	**no**	**yes**	**n**	**no**	**yes**
**Unadjusted model**						
Cuba	220	174	46	222	170	52
Dominican Republic	206	181	25	208	151	57

Peru	137	116	21	137	66	71
Venezuela	178	153	25	181	150	31
Mexico	156	146	10	157	118	39
Puerto Rico	239	223	16	239	198	41
China	204	179	25	204	199	5
**Adjusted model**						
Cuba	206	160	46	208	157	51
Dominican Republic	173	150	23	175	124	51
Peru	134	114	20	134	65	69
Venezuela	159	141	18	162	135	27
Mexico	122	116	6	152	114	38
Puerto Rico	202	188	14	202	164	38
China	202	178	24	189	194	5

Table S4. Cross-tabulation between the number of children (aged under 16 years) in a household and caregiver age

Number of children	Caregiver age		Total
	**Younger caregiver** (%)	**Older caregiver** (%)	(n)
0	61.9	79.0	868
1	19.6	11.5	222
2	11.2	6.2	125
>3	7.3	3.3	79
**Total**	**100.0**	**100.0**	**1 294**
	$\chi^2=36.17$, $p<0.001$		
